# Process Evaluation of a School-Based High-Intensity Interval Training Program for Older Adolescents: The Burn 2 Learn Cluster Randomised Controlled Trial

**DOI:** 10.3390/children7120299

**Published:** 2020-12-16

**Authors:** Sarah G. Kennedy, Angus A. Leahy, Jordan J. Smith, Narelle Eather, Charles H. Hillman, Philip J. Morgan, Ronald C. Plotnikoff, James Boyer, David R. Lubans

**Affiliations:** 1 Priority Research Centre in Physical Activity and Nutrition, School of Education, University of Newcastle, Callaghan, NSW 2308, Australia; sarah.kennedy@newcastle.edu.au (S.G.K.); Angus.Leahy@newcastle.edu.au (A.A.L.); jordan.smith@newcastle.edu.au (J.J.S.); narelle.eather@newcastle.edu.au (N.E.); philip.morgan@newcastle.edu.au (P.J.M.); Ronald.plotnikoff@newcastle.edu.au (R.C.P.); 2 Department of Psychology, Northeastern University, Boston, MA 02115, USA; c.hillman@northeastern.edu; 3 Department of Physical Therapy, Movement and Rehabilitation Sciences, Northeastern University, Boston, MA 02115, USA; 4 New South Wales Department of Education, School Sport Unit, Sydney, NSW 2000, Australia; james.boyer1@det.nsw.edu.au

**Keywords:** implementation, senior students, adolescents, physical activity, health promotion

## Abstract

Process evaluations can help to optimise the implementation of school-based physical activity interventions. The purpose of this paper is to describe the process evaluation of a school-based high-intensity interval training (HIIT) program for older adolescent students, known as Burn 2 Learn (B2L). B2L was evaluated via a cluster randomised controlled trial in 20 secondary schools (10 intervention, 10 control) in New South Wales, Australia. Teachers (*n* = 22 (55% female)) from the 10 intervention schools, delivered the program over three phases (Phases 1 and 2, 6 months; Phase 3, 6 months) to older adolescent students (*n* = 337 (50% female); mean ± standard deviation (SD) age = 16.0 ± 0.4 years). Process evaluation data were collected across the 12-month study period. Teachers delivered 2.0 ± 0.8 and 1.7 ± 0.6 sessions/week in Phases 1 and 2 respectively (mean total 25.9 ± 5.2), but only 0.6 ± 0.7 sessions/week in Phase 3. Observational data showed that session quality was high, however heart rate (HR) data indicated that only half of the students reached the prescribed threshold of ≥85% predicted HR_max_ during sessions. Over 80% of teachers reported they intended to deliver the B2L program to future student cohorts. Almost 70% of students indicated they intended to participate in HIIT in the future. Teachers considered the program to be adaptable, and both students and teachers were satisfied with the intervention. B2L was implemented with moderate-to-high fidelity in Phases 1 and 2, but low in Phase 3. Our findings add to the relatively scant process evaluation literature focused on the delivery of school-based physical activity programs.

## 1. Introduction

Regular physical activity during adolescence is associated with a range of physical [1], psychological [2] and cognitive [3] benefits. Of concern, global estimates suggest only 20% of children and adolescents accumulate the recommended 60 min of moderate- to vigorous-intensity physical activity (MVPA) every day [4,5]. Furthermore, physical activity levels decline dramatically during adolescence [6,7] and only 6% of Australian adolescents (i.e., 15–17 years) achieve the national MVPA guidelines [8,9]. Participation in physical activity, particularly activity of vigorous intensity, is the primary means for improving cardiorespiratory fitness (CRF), which is an important marker of current and future health status [10,11].

Schools are considered ideal settings for the delivery of interventions to combat inactivity and enhance young peoples’ CRF [12], as they have the access to students, facilities, curriculum and staff in which health promotion interventions can be delivered [12]. However, implementing physical activity programs in schools comes with challenges, including teacher confidence and expertise [13,14], competing time pressures [14,15] and school/executive support [14]. Despite their potential, school-based interventions have mostly failed to increase adolescents’ objectively measured MVPA [16,17] and CRF [18]. Of note, larger effect sizes are typically observed in pilot and efficacy studies (typically delivered by researchers) compared to effectiveness and replication studies (usually delivered by teachers) [19]. The success of school-based interventions is largely attributed to the quality of implementation, which can vary considerably in effectiveness and replication studies due to teacher delivery [20,21]. The deviation in implementation (i.e., program drift) that occurs as interventions move from the pilot/efficacy phase to the effectiveness/replication phase typically results in decreasing benefits for participants [22]. Comprehensive process evaluations can help to explain the process of implementation and guide intervention optimisation, defined by Wolfenden and colleagues [23] (pg. 5) as, “a deliberate, iterative and data-driven process to improve a health intervention and/or its implementation to meet stakeholder-defined public health impacts within resource constraints”.

As noted in a recent review by Hung and colleagues [24], the use of an implementation framework is essential for the optimisation of school-based health promotion programs. Frameworks allow researchers and stakeholders to pre-emptively identify barriers that may arise, such as required resources/training and/or support requirements [14,15], as well as pinpoint essential program components [25,26]. In addition to the use of an appropriate framework during planning, it is also important that researchers select necessary outcomes and determinants to evaluate implementation factors [27]. Recently, McKay and colleagues [27] penned an evaluation roadmap, including a minimum set of ten outcomes and five determinants. This guide provides a list of relevant outcomes/determinants, contributing to enhanced implementation evaluation, and the provision of valuable data for both researchers and stakeholders.

Lack of time has emerged as a major barrier to the promotion of physical activity in schools [28]. High-intensity interval training (HIIT) is a time-efficient mode of activity, that consists of short, highly vigorous bouts of activity (e.g., >85% of maximal heart rate (HR)) interspersed with rest or light activity [29]. Numerous systematic reviews have demonstrated the potential for HIIT to improve adolescents’ physical (e.g., CRF, waist circumference), mental (i.e., well- and ill-being) and cognitive (e.g., attention, memory, executive function) health [30,31,32]. In addition to these health benefits, the interval nature of HIIT is also preferred by adolescents, compared with continuous activity (independent of intensity) [33]. Although still emerging, some studies have shown that HIIT can be successfully delivered within the school setting [34], yet the majority of school-based HIIT programs have been delivered by researchers to younger adolescents. However, a common critique of HIIT as a public health approach, is that HIIT interventions have poor reach and high levels of attrition due to feelings of displeasure associated with high-intensity exercise [35]. Although, there is emerging evidence supporting the positive perceptions adolescents have towards HIIT, compared with moderate intensity continuous exercise [36].

**Figure 1:**
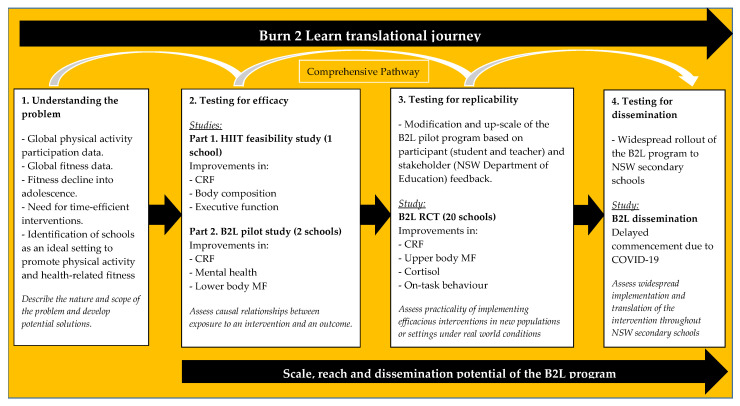
Burn 2 Learn (B2L) research progression model, including pathway to scale-up. Originally adapted from: Milat, Bauman [47] and Nutbeam and Bauman [48].

Burn 2 Learn (B2L) is a school-based physical activity program designed to improve older adolescents’ CRF [37,38,39,40]. The intervention provides students in their final two years of schooling with a new opportunity to be physically active during the school day, via teacher-facilitated HIIT sessions during curricular time. Whilst past school-based HIIT studies have been researcher-led [41,42], teachers were chosen as the delivery agent of the B2L program. This has been done in the majority of school-based physical activity programs to date [21], and presents as a useful strategy to maximise intervention scalability [43]. The B2L program was guided by the Consolidated Framework for Implementation Research (CFIR) [44] and we utilised a range of strategies to ensure the intervention was implemented as intended. We recently evaluated the B2L program in a cluster randomised controlled trial (RCT) in 20 secondary schools with 670 older adolescents [40]. Relative to those in the control group, participants in the intervention group improved their CRF and upper body muscular endurance, and reduced their stress (i.e., assessed using hair cortisol concentrations) [37,40]. We also observed improvements in students’ on-task behaviour and subjective vitality immediately following participation in a B2L session [45], indicating beneficial acute effects. Prior to this effectiveness trial, the B2L program underwent feasibility [42,46] and pilot [38] testing, which represents the first three steps of a ‘comprehensive’ pathway to scale-up (Figure 1). We are currently working with the New South Wales (NSW) Department of Education to implement the program at-scale throughout NSW secondary schools. As such, the primary aim of this paper is to describe the process of implementation of the B2L program in our cluster RCT using McKay and colleagues’ evaluation roadmap [27].

## 2. Materials and Methods

### 2.1. Study Design and Participants

The B2L program was evaluated via a two-arm parallel-group cluster RCT in 20 secondary schools (10 intervention, 10 control) within NSW, Australia. Data collection and intervention delivery occurred via two cohorts of 10 schools (Cohort 1: February 2018–February 2019; Cohort 2: February 2019–February 2020). Schools within each cohort were pair-matched and pairs were randomly assigned to either the B2L intervention or a wait-list control condition (i.e., continued normal school activities) for the 12-month study period. Two teachers from each study school were recruited as school champions (i.e., teachers willing to facilitate intervention delivery). There were no restrictions on the teaching specialisation of school champions. Teachers from schools allocated to the intervention group received training to deliver the B2L program. Eligible participants were Grade 11 students (aged 15–19 years) taught by one of the B2L school champions at the time of baseline assessments. Ethics approval for the B2L RCT was obtained from the Human Research Ethics Committee of the University of Newcastle, Australia (H-2016-0424), and the NSW Department of Education and Communities (SERAP: 2017116). School Principals, teachers, parents and students all provided informed written consent prior to enrolment.

### 2.2. Intervention Description

The B2L intervention was delivered over three phases: Phase 1—*Getting started* (3 months; May–July 2018/19), Phase 2—*Maintaining student interest* (3 months; July–September 2018/19) and Phase 3—*Moving towards independence* (6 months; October 2018/19–February 2019/20). In Phase 1, school champions were instructed to focus on developing students’ HIIT competency and self-efficacy (e.g., developing/reinforcing correct technique and knowledge of HIIT session structure). Phase 2 provided a greater emphasis on student autonomy and responsibility (e.g., providing choice and allowing students to design their own HIIT sessions). During Phases 1 and 2, school champions were instructed to deliver a minimum of two sessions per week during curriculum time. Teachers had the option to choose a session duration ranging from 4 to 16 min (i.e., intervals 1–4 repeated once to four times; see Supplementary Figure S1). In Phase 3, students were encouraged by teachers to complete HIIT sessions outside of school hours (school champions could still facilitate sessions during curriculum time if they desired, however there was no expectation that they do so).

Previous adolescent HIIT studies have utilised highly prescriptive ‘traditional’ HIIT protocols (i.e., singular exercise modes such as running or cycling), as well as expensive and/or specialised equipment (i.e., treadmills, cycles, rowers and step machines) [31]. Additionally, past school-based HIIT interventions have been researcher-led, and delivered to a smaller cohort of students [41,42]. Unlike those prescribed within past studies, B2L sessions were designed to enhance students’ autonomous motivation for exercise [34]. To promote variety and enjoyment, students could select from a number of different themed HIIT workouts, involving a combination of simple aerobic (e.g., shuttle runs, jump rope and dance movements) and muscle-strengthening exercises (e.g., triceps dips, body weight squats and ‘rough and tumble’ activities). A full description of the HIIT sessions are available in our published protocol [37]. The B2L program also included additional intervention/implementation components targeting schools (e.g., equipment and resource pack, facilitator presentation to school staff), teachers (e.g., one-day professional development workshop, two session observations by research team), students (e.g., pre-program interactive seminar, B2L smartphone application) and parents (e.g., video ‘newsletters’ providing information to support student engagement). Of note, a key element of the B2L intervention involved training teachers to facilitate the delivery of school-based HIIT sessions (via the professional learning workshop and facilitator presentation to fellow staff; see B2L protocol paper for further detail [37]). This approach enhances program sustainability and scalability, as it does not rely on research personnel or external providers to deliver sessions, which has been commonplace in prior adolescent HIIT studies.

### 2.3. Theoretical Basis

B2L was guided by self-determination theory (SDT) [49]. Sessions were designed to enhance adolescents’ autonomous motivation for exercise by satisfying their basic psychological needs of autonomy, competence and relatedness [49]. The tenets of SDT were operationalised using the Supportive, Active, Autonomous, Fair and Enjoyable (SAAFE) delivery principles, which provide an evidence-based framework for the delivery of quality physical activity sessions [50]. Sessions were designed to facilitate a supportive environment (e.g., praising student effort and improvement), involve a high level of movement (e.g., session commence quickly, with students working at high-intensity), encourage student autonomy (e.g., students select HIIT type such as Gym HIIT or Sport HIIT), allow all students to experience success (e.g., having multiple challenge levels for exercises) and be enjoyable and engaging for students (e.g., playing motivational music during sessions).

### 2.4. Implementation Framework

**Figure 2:**
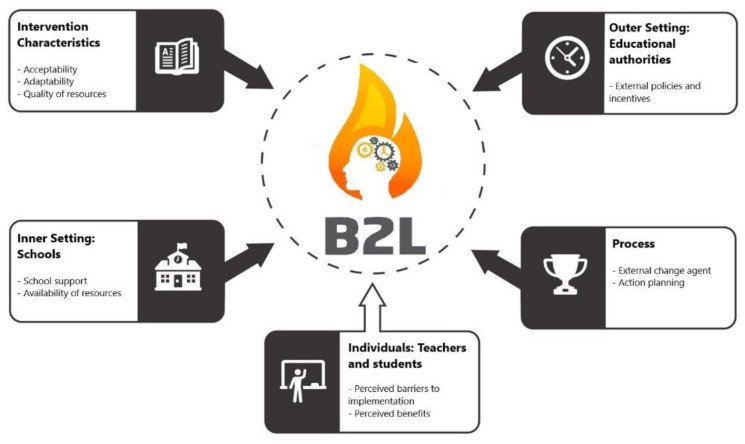
Operationalisation of Consolidated Framework for Implementation Research (CFIR) domains for the B2L intervention.

To support the delivery of the intervention and maximise opportunities for delivery at-scale, the intervention was guided by CFIR [44]. We utilised a range of implementation strategies targeting all five CFIR domains: (i) *Intervention characteristics* (i.e., intervention adaptability and acceptability, design quality and packaging of intervention resources), (ii) *Outer setting* (i.e., external policies and incentives), (iii) *Inner setting* (i.e., identifying barriers to implementation and support), (iv) *Characteristics of individuals* (i.e., knowledge and beliefs about the intervention, benefits for students) and (v) *Implementation process* (i.e., external change agent support and action planning) (see Figure 2).

### 2.5. Process Evaluation

The process evaluation of the B2L program was guided by McKay and colleagues’ [27] minimum dataset (Table 1); Data sources were aligned with the outcomes and determinants presented in Table 1; Implementation of the B2L program was assessed using a variety of methods, including: post-program student and teacher evaluation questionnaires, session observations, session HR monitoring, and consent rates [51].

#### 2.5.1. Implementation Outcomes

*Dose delivered* was assessed via teachers’ logs of the number of sessions delivered over the three phases of the program. Students self-reported how many sessions per week they were completing during Phases 1 and 2, and how often they completed self-directed B2L sessions (i.e., *Never* to *More than three times per week*) during the school holidays. *Reach* was calculated as the proportion of students consenting to participate in the evaluation component of the intervention, compared to the total number of students enrolled in recruited classes. *Fidelity* was operationalised using two separate metrics: (i) *Session quality* was assessed by members of the research team using HIIT session observations conducted twice (per class) during the intervention period (approximately weeks 4 and 8). Researchers evaluated session quality using a four-point scale (i.e., 1 = *Strongly disagree* to 4 = *Strongly agree*), with items corresponding to the presence (or lack thereof) of suggested strategies for satisfying the SAAFE delivery principles (see Supplementary Figure S2 for lesson observation template). (ii) *Session intensity* was assessed using HR monitors during HIIT sessions. All students were provided with a Wahoo TICKR HR monitor and were instructed to wear their device during sessions. The HR monitor connected via Bluetooth to the purpose-built B2L smartphone and tablet application (app) during sessions to collect student HR data. Mean peak HR and mean HR for the entire session (both expressed as % HR_max_) were calculated to determine whether participants exercised at the recommended intensity (i.e., ≥85% predicted HR_max_). Number of students achieving a peak, or mean, HR at ≥85% predicted HR_max_ were also calculated. *Sustainability* was assessed via teacher and student post-program evaluation questionnaires. Teachers reported their intention (Yes/No) to deliver B2L in the future (sustainability). Students reported their intention (Yes/No) to participate in HIIT in the two months following program completion.

#### 2.5.2. Implementation Determinants

The implementation determinants presented in Table 1 were collected via the teacher and student post-program evaluation questionnaires. Teachers responded to questions related to each determinant on a four-point scale (i.e., 1 = *Strongly disagree* to 4 = *Strongly agree*). Students responded to the satisfaction question on a five-point scale (i.e., 1 = *Poor* to 5 = *Excellent*). Students and teachers also had the opportunity to provide comments (positive and negative) and suggestions for improvement when completing their post-program evaluation questionnaires. These comments were assessed for suitability related to each of the included determinants and reported where appropriate.

## 3. Results

A total of 10 schools (*n* = 337 students (50% female), *n* = 22 teachers (55% female)), were allocated to receive the intervention. Characteristics of the consenting students and included schools are presented in Table 2. Three quarters of intervention students were of low-to-medium socioeconomic status, with 7% of Indigenous heritage. Just over 70% of B2L students reported an Australian cultural background. Intervention schools had close to a 1000 enrolments per school, with a higher proportion of females than males. Twenty percent of schools were selective, whilst the majority were located in major cities and included students from grades 7–12. Given that there were no restrictions on the teaching specialisation of school champions, the 22 intervention classes included in the study were made up of: Biology = 1, Community and Family Studies = 1, Mathematics = 2, Modern History = 1, Health and Physical Education = 13, Sports Coaching = 1, and Sport Leisure and Recreation = 3. The B2L intervention was delivered during curriculum time of these specific subjects, relevant to the included class. All but one teacher (*n* = 21) completed the post-program evaluation questionnaire, as this teacher was unable to be contacted at follow-up to compete the evaluation. Of the 22 teachers, all facilitated at least one SAAFE session observation (conducted by the research team), with 15 teachers (68%) facilitating two observations. In addition, 267 (79%) students completed the post-program evaluation questionnaire.

### 3.1. Implementation Outcomes

The results for implementation outcomes are presented in Table 3.

*Dose delivered:* Teachers delivered an average of 2.0 ± 0.8 B2L sessions during Phase 1 (the minimum requirement) and 1.7 ± 0.6 in Phase 2 (below the minimum requirement), with a range of 0 to 3 sessions per week. Students reported a mean of 3.2 ± 0.9 sessions completed each week during Phases 1 and 2. Almost 42% of students reported that they had completed B2L sessions during the two-week school holiday period (between Phases 1 and 2). Sixteen percent reported less than one session per week (i.e., one session over the whole holiday period), 10% reported one session per week, 11% two sessions per week and 5% three or more sessions per week. In Phase 3, half of the teachers reported delivering between 0 and 2 sessions per week, with 0.6 ± 0.7 sessions per week delivered on average. The mean number of reported sessions (during Phases 1 and 2, combined) was 25.9 ± 5.2 per class, the majority of which were 8 min in duration. *Reach:* Almost 80% of students in recruited classes consented to participate in the program evaluation. *Fidelity: Session quality*—Overall, researcher fidelity observations showed B2L sessions were delivered as intended (16.0/20.0 ± 2.6 units). We observed a slight increase in overall session quality between the first and second observations. The *Supportive* and *Fair* teaching principles were implemented most effectively by teachers. During the first observation, teachers utilised strategies related to three out of five of the SAAFE principles in their lessons. Based on scores below 3 (i.e., *Agree*) for the *Autonomous* and *Enjoyable* principles, it was evident they were not addressed as effectively as others. During the second observation, session quality scores increased for the majority of indicators, however scores for the *Autonomous* principle still scored below 3. *Session intensity:* Data from HR monitoring (collected via the B2L app) showed the mean HR across all sessions was 70% ± 11% HR_max_, whereas the mean peak HR across all sessions was 82% ± 10% HR_max_. Half of the students reached a peak HR_max_ ≥ 85% during the sessions, whilst only 17% achieved a mean HR_max_ ≥ 85% over the entire session. *Sustainability:* Over 80% of teachers reported that they would deliver the program to future groups of students. Almost 70% of students reported that they intended to continue participating in HIIT over the next two months.

### 3.2. Implementation Determinants

Findings for included implementation determinants are presented in Table 4 and Table 5.

*Context:* Teachers were relatively neutral about whether departmental endorsement influenced their choice to implement the intervention (2.6 ± 0.8 units). *Acceptability:* Teachers agreed the quality and design of the resources were acceptable (3.1 ± 0.6 units). However, teachers agreed less that the time required to deliver B2L sessions was acceptable (2.3 ± 0.6 units). Teachers provided multiple comments related to intervention acceptability. *Adaptability:* Adaptability of the B2L intervention to the school was found to be high (3.4 ± 0.6 units). *Compatibility:* Teachers perceived that the intervention was successful in improving students’ on-task behaviour (3.2 ± 0.6 units), academic performance (3.3 ± 0.7 units) and mental health (3.6 ± 0.5 units). Teachers provided multiple comments related to intervention compatibility. *Culture:* Teachers agreed fellow staff (3.4 ± 0.6 units) and school executives (3.3 ± 0.7 units) were supportive of the B2L program. *Dose (satisfaction):* Overall program satisfaction was high for teachers (3.3 ± 0.5 units) and moderate-to-high for students (3.8 ± 0.9 units). Teachers were also satisfied with the support provided by the research team (3.6 ± 0.5 units). Teachers and students provided multiple comments related to satisfaction. *Complexity:* Relative difficulty of the intervention was low, as teachers reported ease of implementation (3.0 ± 0.7 units). Relevant comments related to intervention complexity were provided by teachers. *Self-efficacy:* Teachers were highly confident in their ability to deliver the B2L program (3.5 ± 0.6 units). One teacher provided a comment related to self-efficacy.

## 4. Discussion

The aim of this study was to describe the process evaluation of a school-based high-intensity interval training (HIIT) program for older adolescent students called B2L, utilising outcomes and determinants from McKay and colleagues’ framework [27]. Findings indicate the B2L program was successfully implemented in Phases 1 and 2, with positive findings in terms of reach, fidelity and intention to deliver/participate in the future. There were also positive findings for a range of implementation determinants, including teachers’ perceptions of program adaptability, compatibility, satisfaction and self-efficacy. Of note, teachers reported some concerns regarding the time required to the deliver the program.

Considering the scarcity of research done with older adolescents, and given the unique challenges of working with this population, it was promising to find teachers delivered close to the prescribed number of sessions (two/week, 28 total) during Phases 1 and 2 [37]. Teacher-selected HIIT session durations varied between teachers, ranging from 4 to 16 min. Despite this variation in duration, fidelity was maximised through the use of the B2L task cards, which provided teachers/students to modify the number of rounds they completed (see Supplementary Figure S1). Specific options for HIIT session duration, and variety in session type (i.e., different HIIT workouts), allowed for teachers to adapt the program to their school and students [52]. This was well-received by teachers, with positive reports regarding their ability to adapt B2L based on individual school characteristics. Previous studies have shown the importance of providing options surrounding session delivery time [53], highlighting the need for intervention delivery flexibility. Further, to enhance the likelihood of program success and continued delivery into the future, there is a need for researchers to create scalable programs that can be adapted to various contexts [43,54]. Despite the presence of adaptability, programs should include core components [55]. These are the elements that have been identified to contribute most to program effectiveness [55]. For B2L, the focus on HIIT and delivery of two sessions per week represent core components. Conversely, session type and duration are peripheral inclusions to increase engagement through providing options for varying student abilities and interest, as well as differing environments. Whilst the core goal of two sessions per week during Phases 1 and 2 was not quite met, the finding that the majority of teachers were following (or exceeding, i.e., three sessions) was promising. Identifying core components and instilling their importance within delivery agents through training, whilst also preparing for adaptation of the peripheral components, is an important step in minimising the effect of a trade-off between fidelity and adaption [56].

The crowded curriculum is a considerable barrier to the implementation of physical activity interventions in schools [57], especially during the senior years. As such, teachers’ perceptions of the acceptability of intervention delivery time presents some concern. Interestingly, the 8-min sessions were most popular, whilst the 16-min were the least (only one reported across intervention schools). With set-up, session delivery and transition back to the academic lesson, 8-min sessions were completed in ~15 min. Applying this same logic, the 16-min session may have taken up to 30 min in total, which was likely no longer categorised as ‘time-efficient’ (as some lessons may be as short as 40 min). Given the popularity of the 8-min sessions, 15 min may be the maximum amount of time teachers were willing to take out of lesson time to maintain the allure of time-efficiency of HIIT. These findings could provide guidance for the design and implementation of future school-based interventions. Finding an acceptable session duration, including preparation and pack up time, is imperative to maximising implementation success. Lack of time is the major barrier to the implementation of school-based physical activity interventions [14,15]. Conversely, scheduling (i.e., allocating specific time for) physical activity opportunities, facilitates implementation [14]. The importance of scheduling is evident in our trial, as few sessions were delivered by teachers in Phase 3. Although teachers were given the option to continue session delivery in Phase 3 (Moving towards independence), and half did so, the focus was on encouraging students to complete sessions in their own time. Despite the adaptability of the program, and adherence during the first two phases, once given the option, fewer teachers implemented the sessions and there is little evidence that students completed the sessions outside of school time during Phase 3. As such, future research aiming to include additional opportunities for MVPA during the school day will benefit from educating delivery agents (such as teachers), school administrators and executive staff on the importance of including activity opportunities in the weekly timetable and keeping this within their schedule. These timetabled activity breaks provide an expansion on what is currently being offered for older adolescents in NSW (and many others in their senior schooling years), replacing traditional sedentary lesson time with scheduled occasions to participate in MVPA [58].

The provision of teacher training (i.e., professional learning workshop and research team support) is essential for high-quality implementation [15,52]. Professional learning was provided to teachers prior to delivery of the B2L intervention, to increase their knowledge, skills and confidence to implement the program. Specifically, B2L school champions were educated on the SAAFE principles [50] and ways in which these could be incorporated into lessons. In addition to this initial training, teachers also received two session observations and feedback, and ongoing research team support. Based on our observations, it was evident that teachers had gained knowledge and skills related to the incorporation of SAAFE strategies into their lessons. This was apparent due to the high levels of adherence to principles in both observation 1 and 2, with a slight improvement over time. In particular, improvements for the *Autonomous* and *Enjoyable* principles were notable during the second observation. This suggests teachers were providing students with additional choice (*Autonomous*) and enjoyment/variety (*Enjoyable*) during sessions. These improvements may have been in part due to feedback offered by the research team following the first observation [59], whereby researchers offered suggestions of ways in which to improve sessions, and fielded any questions from the teacher. Further, ongoing support provided by the research team may have helped increase teacher capability and understanding on ways in which to deliver ‘SAAFE’ sessions. Positive feedback provided by teachers related to research team support backs this notion, however future research may benefit from looking at how variations in support may impact implementation quality.

Teachers were also provided with information surrounding the benefits of HIIT training [30,31,32,38]. Knowledge of the benefits of HIIT, along with continued program delivery as the intervention proceeded, may have had bearing on teachers’ beliefs surrounding the appropriateness of the program within the school context. As schools’ core business is teaching and learning [60], the ability to link physical activity participation with improvements in on-task behaviour and academic performance may provide researchers with added justification to deliver physical activity programs. To provide evidence for the academic benefits of the B2L program, we conducted a sub-study in 10 schools and *n* = 221 students [45]. We observed significant group-by-time effects in students’ on-task behaviour and subjective vitality [45]. In addition to these findings, it was also promising that during post-program evaluation, teachers perceived B2L to be of benefit to students’ mental health, academic performance and on-task behaviours. These perceptions support sub-study findings [45], and provide potential reasoning for the high-quality implementation of B2L. Teachers believed the intervention to be an appropriate tool to reach their goals (i.e., enhanced teaching and learning) and of benefit their students.

Regarding session intensity, half of the students achieved a peak HR at the intended threshold (i.e., ≥85% HR_max_) during B2L sessions and the mean peak HR was below this threshold (82% HR_max_). A previous school-based study [41] has shown that researcher-led sessions can yield a mean peak HR of 85% HR_max_ (when considering intention-to-treat), so it was promising that the mean peak HR_max_ was only slightly lower in the teacher-led B2L sessions. Importantly, the efficacy of HIIT for optimising fitness and related health outcomes is contingent on participants performing exercises at a high intensity (i.e., ≥85% HR_max_). Although some health benefits are no doubt achieved by exercising at lower (but still vigorous, i.e., ≥70% HR_max_) intensity [61], the potency of HIIT is likely reduced as intensity declines. As such, sessions may need to be extended for individuals to attain similar benefits. This is problematic, as time is a legitimate barrier to the delivery of HIIT in schools, and especially among this population of students (as we also found). However, it may be unrealistic to expect students that are not necessarily motivated to exercise at high intensity for an extended period of time. Having said this, our published outcomes paper [40] identified a number of meaningful intervention effects, despite HIIT session intensity being lower than intended. Our research demonstrates both the exercise intensity that can be expected when translating HIIT into a real-world school context, and the outcomes that can be achieved with this level of exercise intensity. In contrast, when delivered in laboratory settings, participant to researcher ratio is lower and specialised equipment is available. Previous school-based studies have also been delivered to a smaller cohort of students by researchers [41,42]. Conversely, the B2L intervention was delivered by teachers (not researchers) to a class of students (i.e., up to 25 students) with little to no equipment. These sessions were also designed to be enjoyed by students and enhance their autonomous motivation for vigorous physical activity. As such, this finding suggests researchers may face a trade-off between rigid protocols (i.e., HR > 85%) and what is a realistic expectation for delivery in schools. Whilst still benefiting from the increased intensity and time-efficiency HIIT affords, it is necessary to ensure intervention flexibility in order to allow delivery across a variety of school contexts, while still maintaining appropriate HIIT protocols [29]. Of note, the varied session types may have influenced the intensity of the session, however this was not assessed. Evaluation of the impact of session type on recorded intensity may provide information for the design and development of future programs.

The provision of resources to schools, including equipment and resource packs, and access to the B2L smartphone/tablet app, was provided in an effort to support program delivery and align with HIIT conventions (i.e., inbuilt work rest timer within the app, including exercise images and GIFs and a visual HR display when wearing a TICKR HR monitor). Resources allowed students to design their own sessions (i.e., competency and autonomy), and to develop their exercise skills (i.e., competency) using technique cards. The provision of resources has also been identified as a facilitator of quality implementation [14], so positive findings surrounding teachers’ perceptions of the resources is promising. These resources may aid in the delivery of sessions, and work towards maximising the potential of high-quality school-based HIIT sessions to be delivered at an intensity nearing the >85% threshold.

Almost 70% of students reported their intention to participate in HIIT over the next two months. However, findings for CRF at 12 months [40] may be in part explained by students not engaging in self-directed HIIT sessions. Once the structured curriculum-time sessions were discontinued after Phases 1 and 2, students’ CRF improvements were lost [40]. This may be in part explained by the ‘Structured Days Hypothesis’ [62], whereby the structure provided within the school day protects students from physical inactivity. It is important to note that our intervention was guided by the theory of expanded, extended and enhanced opportunities for youth physical activity [58]. As such, we hypothesised that the removal of the opportunity would lead to decreased participation and fitness, despite students’ intention, satisfaction with the program and improvements in HIIT self-efficacy [40]. Student HIIT self-efficacy may provide reasoning for their satisfaction and engagement with B2L during Phases 1 and 2 [63]. However, without the planned nature of teacher-facilitated sessions seen in Phases 1 and 2, intentions to continue participation were unlikely translated into behaviour [64]. This finding provides valuable insight into the need for the provision of organised, embedded, physical activity opportunities for all (not just older) adolescents. We urge future research and policy to consider this in design, development and decision-making processes. As, despite best intentions in past school-based studies to improve adolescent behaviour, findings have been modest [65]. These opportunities have the potential to improve not only school-based physical activity but may also have a positive impact on overall physical activity behaviour of adolescents.

Interestingly, over 80% of teachers also reported intention to deliver B2L to future student cohorts. While this is not a true indicator of sustainability, it does at least suggest endorsement of the program from participating teachers. Possible reasoning for this level of intention from students and teachers may be in part due to the favourable findings for self-efficacy and program satisfaction. However, self-reported intentions are a limited indicator of sustainability and future research will benefit from a more robust measure of sustainability.

An important factor that may have influenced implementation success is the perception amongst teachers that fellow staff and school executive were supportive of the B2L program. Limited support from school executive staff has previously been cited as one of the major barriers to implementation of school-based interventions [13,14]. As such, positive findings for school culture were promising. One improvement that could be noted though, is the lack of recognition teachers felt as a result of delivering B2L. Previous research cited the involvement from leaders (i.e., school executive) as a facilitator of quality implementation [52,66]. This involvement includes their engagement with the program, as well as their recognition of staff contributions. However, it is interesting that the teacher’s reports of his feeling of limited support was not echoed in findings for context.

### Strengths/Limitations

Strengths of this study include alignment with accepted outcomes and determinants to describe implementation, and our comprehensive process evaluation utilising various sources. There are some limitations that should be noted. First, not all sessions were conducted with HR monitors. Reported HR data is representative of sessions where students wore HR monitors. Second, the majority of implementation outcome and determinant data were self-reported by teachers, which are prone to social desirability bias. Indeed, the self-report data on intention to continue delivering HIIT, and the Phase 3 teacher-reported session delivery rate were somewhat discrepant, perhaps suggesting social desirability bias contributed to teachers’ responses on this item. Additionally, we do not have objective data on the number of sessions completed by students in Phase 3. Finally, the majority of classes that agreed to participate in the B2L study were Health and Physical Education classes, which limits the generalizability of our findings. Future research would benefit from the ability to assess program success across a more representative sample of students and ascertain any differences that may exist across subject areas.

## 5. Conclusions

The B2L intervention was successfully implemented in a sample of secondary schools, with a host of positive program-related perceptions reported by teachers. Our findings add necessary process evaluation data to the literature, highlighting the need to assess the way in which implementation may impact findings for primary and secondary outcome measures. Outcomes from this study provide recommendations regarding the need for ongoing support for both students and teachers, and the embedding of activity opportunities within the school day, to maximise implementation quality and sustained participation. This study also provides information related to variations from standard, laboratory based HIIT protocols that may be necessary when delivering in real-world settings (i.e., schools), guiding future researchers on potential adaptations and inclusion that may be necessary to increase implementation potential. The need for expanded physical activity opportunities for older adolescents is also highlighted, as a means to overcome issues surrounding behaviour change and exercise self-regulation. More research is needed to investigate how the variations in session intensity may affect improvements in CRF, and how to maximise department- and school-level support and recognition for teachers.

## Figures and Tables

**Table 1:** Operationalisation of implementation outcomes and determinants.

		McKay and Colleagues’ [27] Definition	Operationalisation within B2L
*Outcomes*	**Dose delivered**	Intended units of each intervention component delivered to participants by the delivery team	(a) Number of B2L sessions delivered by teachers, throughout the three phases of delivery. (b) Student-reported session participation (c) Proportion of students completing self-directed sessions during the school holidays
	**Reach**	Proportion of the intended priority audience (i.e., participants) who participate in the intervention	Proportion of students from classes involved in the intervention that consented to participating
	**Fidelity**	The extent to which an intervention is implemented as it was prescribed in the intervention protocol—by the delivery team	(a) Session quality (as delivered by teachers) was assessed by members of the research team, utilising the SAAFE observation checklist (see Supplementary Figure S2). (b) Session intensity was also assessed throughout the intervention, using HR monitors.
	**Sustainability**	Whether an intervention continues to be delivered	(a) Teachers’ intention to deliver the B2L program to future student cohorts (b) Students’ intention to participate in HIIT
*Determinants*	**Context**	Aspects of the larger social, political and economic environment that may influence intervention implementation	Impact of the NSW Department of Education professional learning requirements and endorsements on implementation
	**Acceptability**	Perceptions among the delivery team that a given intervention is agreeable, palatable, or satisfactory	Teachers’ perceptions of the: (a) Quality of program resources (b) Acceptability of intervention delivery time
	**Adaptability**	Extent to which an intervention can be adapted, tailored, refined, or reinvented to meet local needs	Teachers’ perceptions of the adaptability of the program dependent on school/class characteristics
	**Compatibility**	Extent to which an intervention fits with the mission, priorities and values of organisations or settings	Teachers’ perceptions of increases in students’ on-task behaviour, academic performance and mental health
	**Culture**	Organisations’ norms, values and basic assumptions around selected health outcomes	(a) School executives and teacher support of the B2L program (b) Teacher provided with recognition for delivering the program
	**Dose (satisfaction)**	Delivery team’s satisfaction with an intervention and with interactions with the support system	(a) Teachers’ overall program satisfaction (b) Students’ overall program satisfaction (c) Interactions with external change agents
	**Complexity**	Perceptions among the delivery team that a given intervention is relatively difficult to understand and use; number of different intervention components	Teachers’ perceptions of program implementation
	**Self-efficacy**	Delivery team’s belief in its ability to execute courses of action to achieve implementation goals	Teachers’ perceptions of implementation capability

Abbreviations: B2L, Burn 2 Learn; SAAFE, Supportive, Active, Autonomous, Fair, Enjoyable; HIIT, high-intensity interval training.

**Table 2:** Characteristics of B2L Students and Schools.

Characteristics		B2L Intervention Schools (*n* = 10)	Control Schools (*n* = 10)
**Student**	Socioeconomic status, mean (SD) %; range ^a^ Low Medium High	81 (24.3) 169 (50.8) 83 (24.9)	48 (14.5) 170 (51.4) 113 (34.1)
	Indigenous decent, *n* (%) ^b^ Yes No	24 (7.2) 311 (92.8)	37 (11.2) 294 (88.8)
	Cultural background, *n* (%) ^b^ Australian European African Asian Middle Eastern Other	239 (71.3) 39 (11.6) 3 (0.9) 21 (6.3) 4 (1.2) 29 (8.7)	230 (69.5) 28 (8.5) 3 (0.9) 18 (5.4) 3 (0.9) 49 (14.8)
**School**	Student enrolments Total enrolments, N Female enrolments, *n* (%) Male enrolments, *n* (%) Enrolments per school, mean (SD); range Female enrolments per school, mean (SD); range Male enrolments per school, mean (SD); range	9634 5524 (57) 4210 (43) 963 (329); 588–1575 552 (316); 289–1322 421 (259); 0–1019	9675 4623 (48) 5052 (52) 966 (306); 600–1426 462 (137); 294–694 505 (171); 306–732
	Selective schools, *n* (%) Academic, *n* (%) Performing Arts, *n* (%)	2 (20) 1 (10) 1 (10)	1 (10) 1 (10) 0 (0)
	School location, *n* (%) Major Cities Inner Regional	9 (90) 1 (10)	10 (100) 0 (0)
	School type, *n* (%) 7–12 K-12 11–12 10–12	8 (80) 1 (10) 0 (0) 1 (10)	8 (80) 0 (0) 2 (20) 0 (0)
	School Index of Community Socio-Educational Advantage (ISCEA) ^c^ ISCEA Mean (SD); Range ^d^ ISCEA Percentile Mean (SD) ^e^; Range	998 (86); 871–1120 47 (34); 5–90	1020 (83); 929–1153 54 (33); 15–96

^a^ Socioeconomic status determined by population tertile using socio-economic indexes for areas of relative socioeconomic disadvantage based on residential postcode; six participants did not provide their residential postcode. ^b^ Four participants (two intervention, two control) did not answer the background demographic questions ^c^ This score is derived from a number of variables including parental school and non-school education and occupation, the school’s geographical location and proportion of Indigenous students; 1000 is average. ^d^ One control school did not report the ISCEA value. ^e^ The percentile of the school’s ICSEA value. One intervention and two control schools did not report the ISCEA percentile. SD: standard deviation.

**Table 3:** Findings for the included implementation outcomes.

**Dose delivered**		Teacher reported B2L sessions/week, mean (SD) Teacher reported B2L sessions/week, range Proportion of teachers delivering sessions during each Phase, % (*n*)	*Phase 1* 2.0 (0.8) 0–3 90 (19)	*Phase 2* 1.7 (0.6) 0–3 95 (20)	*Phase 3* 0.6 (0.7) 0–2 50 (10)
		Total number of teacher reported B2L sessions delivered/week), mean (SD) Typical length of teacher reported B2L sessions, *n* (%) 4 min 8 min 12 min 16 min Student reported participation in B2L sessions/week, mean (SD) Students completing B2L session in the school holidays, *n* (%) Never <1/week 1/week 2/week 3/week More than 3/week	25.9 (5.2) 4 (19.0) 12 (57.1) 4 (19.0) 1 (4.8) 3.2 (0.9) 154 (57.7) 43 (16.1) 26 (9.7) 30 (11.2) 9 (3.4) 5 (1.9)		
**Reach**		Consent rates from intervention schools, mean % (SD)	79.0 (18.2)		
**Fidelity**	*Session Quality*	Adherence to SAAFE delivery principles, mean (SD) Supportive (/4) Active (/4) Autonomous (/4) Fair (/4) Enjoyable (/4) Session total (/20)	*Observation 1* 3.5 (0.7) 3.1 (0.8) 2.5 (0.9) 3.3 (0.8) 2.8 (1.1) 15.1 (3.2)	*Observation 2* 3.5 (0.7) 3.3 (0.7) 2.9 (1.1) 3.5 (0.6) 3.2 (0.9) 16.3 (2.7)	*Overall* 3.5 (0.6) 3.3 (0.7) 2.8 (0.9) 3.4 (0.6) 3.1 (0.8) 16.0 (2.6)
	*Session Intensity*	Average HR during sessions, mean beats per minute (SD) Average HR during sessions, mean % of HR_max_ (SD) Number of students achieving Average HR ≥ 85% HR_max_, n (% whole cohort) Peak HR during sessions, mean beats per minute (SD) Peak HR during sessions, mean % of HR_max_ (SD) Number of students achieving Peak HR ≥ 85% HR_max_, n (% whole cohort)	143.1 (21.8) 70 (11) 56 (17) 167.6 (20.4) 82 (10) 170 (50)		
**Sustainability**		Intention to deliver to future cohorts of students (Yes, %) Student intention to participate in HIIT in the future (Yes, %)	81.8 69.6		

Abbreviations: SAAFE, Supportive, Active, Autonomous, Fair, Enjoyable; HR, heart rate.

**Table 4:** Means of, and qualitative findings for, the included implementation determinants.

Determinant	Evaluation Questionnaire Item	Mean (SD)	Qualitative Comments from Teachers ^a^/Students ^b^
Context ^c^	(a) Implementation facilitated to satisfy professional learning requirements	2.0 (0.8)	NC
	(b) Implementation facilitated due to endorsement by the NSW Department of Education	2.6 (0.8)	NC
Acceptability ^c^	(a) Quality and acceptable design of resources	3.1 (0.6)	(1) Great resources with support cards and music! (2) The resources provided for the B2L program were very useful. (3) I enjoyed the B2L program and believe the resources were great.
	(b) Acceptability of intervention delivery time	2.3 (0.6)	(1) Sometimes the time including transition, etc. took a little longer than I had hoped. (2) Around assessment periods it was harder to implement and find time to complete.
Adaptability ^c^	Adaptation to school characteristics	3.4 (0.6)	NC
Compatibility (Appropriateness) ^c^	*Perceived improvements in student:*		
	(a) On-task behaviour	3.2 (0.6)	(1) Each student who participated enjoyed the sessions and were more alert and could concentrate more in class after the session. (2) Benefit to students.
	(b) Academic performance	3.3 (0.7)	(1) Each student who participated enjoyed the sessions and were more alert and could concentrate more in class after the session. (2) Benefit to students.
	(c) Mental health	3.6 (0.5)	(1) Benefit to students.
Culture ^c^	(a) Teachers at school supportive of B2L	3.4 (0.6)	NC
	(b) School executives supportive of B2L	3.3 (0.7)	NC
	(c) Incentives and recognition for implementing B2L	2.3 (0.7)	NC
Dose (Satisfaction)	(a) Teacher satisfaction, mean (SD) ^c^	3.3 (0.5)	(1) B2L was a great success. (2) I enjoyed the B2L program. (3) Personally, I enjoyed the program and definitely support the reasoning behind the implementation of physical activity in senior years.
	(b) Student satisfaction, mean (SD) ^d,^	3.8 (0.9)	(1) Everything, it was all heaps good. (2) It was very enjoyable and great to do with friends. (3) Encouraged me to exercise as it was a part of class and I had time to participate
	(c) Research team support, mean (SD) ^c^	3.6 (0.5)	(1) The knowledge and support provided by the University were excellent. (2) I liked that the University were in touch constantly. (3) The support when the uni attended was beneficial.
Complexity ^c^	Ease of implementation	3.0 (0.7)	(1) Easy to implement on a weekly basis. (2) The program was easy to implement.
Self-efficacy ^c^	Teacher confidence to implement B2L	3.5 (0.6)	(1) Great program I was really excited to implement.

Abbreviations: SD, standard deviation; NC, no relevant comments; ^a^ Teacher provided comments and improvement suggestions. ^b^ Student provided comments (both positive and potential improvements). ^c^ Measured on a 4-point scale, ranging from *Strongly disagree* (1) to *Strongly agree* (4). ^d^ Measured on a 5-point scale, ranging from *Poor* (1) to *Excellent* (5).

**Table 5:** Frequencies of reported implementation determinants.

	Determinant	Evaluation Questionnaire item	SD (*n*, %)	D (*n*, %)		A (*n*, %)		SA (*n*, %)
**Teacher**	Context ^a^	(a) Implementation facilitated to satisfy professional learning requirements (b) Implementation facilitated due to endorsement by the NSW Department of Education	6 (29) 1 (5)	9 (43) 9 (43)		6 (29) 8 (38)		0 (0) 3 (14)
	Acceptability ^a^	(a) Quality and acceptable design of resources (b) Acceptability of intervention delivery time	0 (0) 1 (9)	2 (10) 6 (55)		14 (67) 4 (36)		5 (24) 0 (0)
	Adaptability ^a^	Adaptation to school characteristics	0 (0)	1 (5)		10 (48)		10 (48)
	Compatibility (Appropriateness) ^a^	*Perceived improvements in student:* (a) On-task behaviour (b) Academic performance (c) Mental health	0 (0) 0 (0) 0 (0)	2 (10) 2 (10) 0 (0)		13 (62) 10 (48) 9 (45)		6 (29) 9 (43) 11 (55)
	Culture ^a^	(a) Teachers at school supportive of B2L (b) School executives supportive of B2L (c) Incentives and recognition for implementing B2L	0 (0) 1 (5) 2 (10)	1 (5) 0 (0) 11 (52)		11 (52) 11 (52) 7 (33)		9 (43) 9 (43) 1 (5)
	Dose (Satisfaction)	(a) Teacher satisfaction, mean (SD) ^a^	0 (0)	0 (0)		14 (67)		7 (33)
		(b) Research team support, mean (SD) ^a^	0 (0)	0 (0)		8 (38)		13 (62)
	Complexity ^a^	Ease of implementation	0 (0)	5 (24)		10 (48)		6 (29)
	Self-efficacy ^a^	Teacher confidence to implement B2L	0 (0)	1 (5)		9 (43)		11 (52)
**Student**	Dose (Satisfaction) ^b^		P	F	Av		G	E
		Student satisfaction, mean (SD) ^b^	7 (3)	15 (6)	50 (19)		137 (51)	58 (22)

Abbreviations: SD, strongly disagree; D, disagree; Ag, agree; SA, strongly agree; P, poor; F, fair; Av, average; G, good; E, excellent; ^a^ Measured on a 4-point scale, ranging from *Strongly disagree* (1) to *Strongly agree* (4); ^b^ Measured on a 5-point scale, ranging from *Poor* (1) to *Excellent* (5).

## Supplementary Materials

The following are available online at https://www.mdpi.com/2227-9067/7/12/299/s1.
