# Supplementary material for: Process Evaluation of a School-Based High-Intensity Interval Training Program for Older Adolescents: The Burn 2 Learn Cluster Randomised Controlled Trial
Source: Children (Basel). 2020 Dec 16;7(12):299. doi: 10.3390/children7120299 (PMC7765884; doi:10.3390/children7120299)
Supplement: Supplementary file 1 [file children-07-00299-s001.pdf]

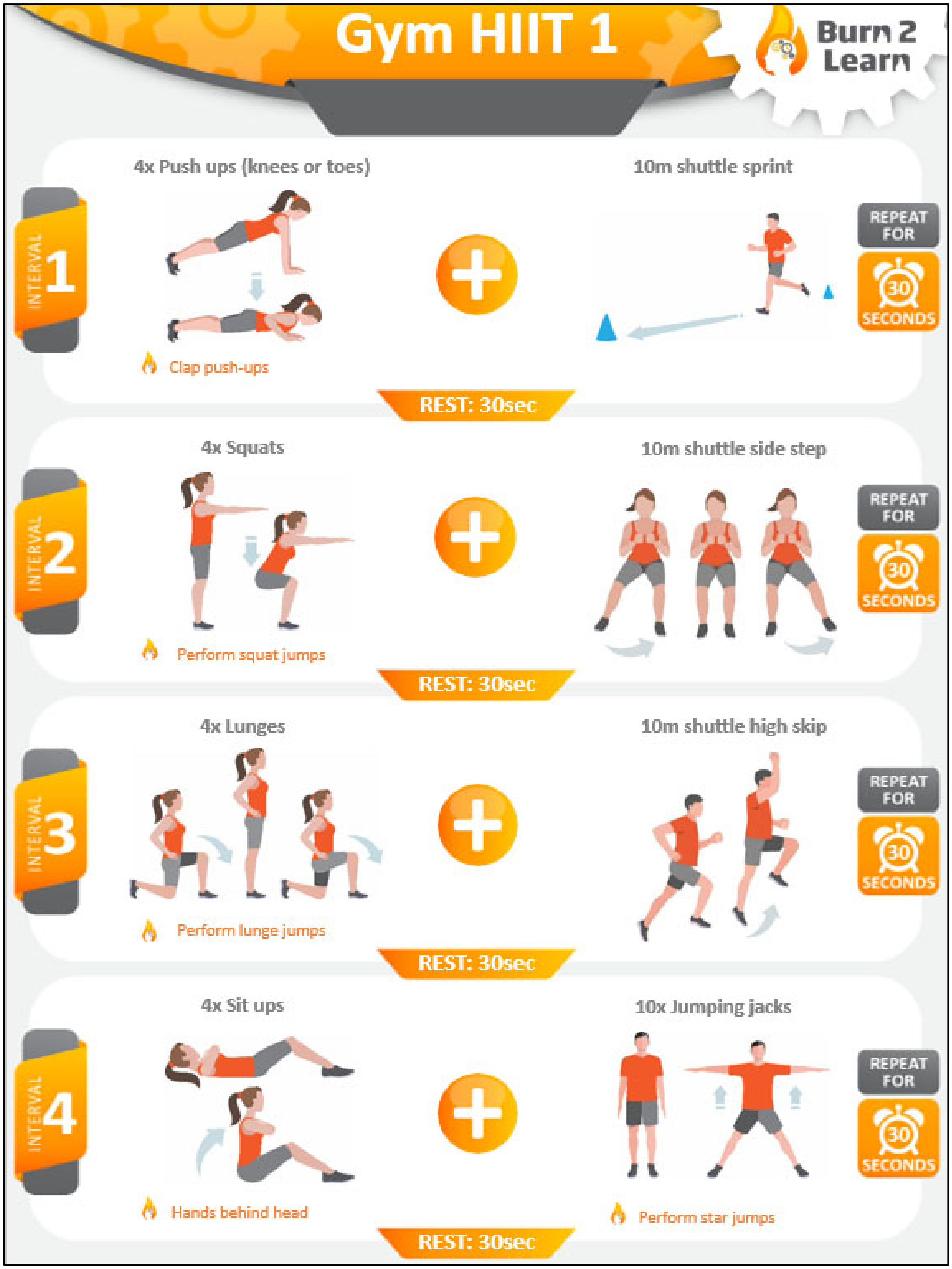

Supplemental Figure 1. Example B2L Task Card.

Burn 2 Learn

SAAFE Principles Observation Checklist

THE UNIVERSITY OF NEWCASTLE AUSTRALIA

ACU AUSTRALIAN CATHOLIC UNIVERSITY

	Adherence to principle			
Teacher:				
Date: School:	Strongly disagree	Disagree	Agree	Strongly agree
	(circle one response)			
S Supportive — SUPPORTIVE: Teacher was supportive and promoted positive student interactions:<br>• Provide constructive feedback<br>• Praise student effort and improvement<br>• Encourage supportive behaviour among students (e.g., high fives)	1	2	3	4
	(circle one response)			
A Active — ACTIVE: Session was highly active and transition time was minimal:<br>• Commence session quickly<br>• Minimise talk and instruction time<br>• Encourage students to exercise at a high intensity	1	2	3	4
	(circle one response)			
A Autonomous — AUTONOMOUS: Session involved opportunities for student choice:<br>• Provide students with opportunities for choice (e.g., music, partner, activity)<br>• Minimise controlling language (e.g., ordering students around)<br>• Remind students about the benefits of high intensity activity	1	2	3	4
	(circle one response)			
F Fair — FAIR: All students provided with opportunities to experience success:<br>• Encourage self-comparison rather than peer-comparison<br>• Encourage students to modify exercises to personal fitness and ability level<br>• Treat all students equally and fairly (i.e., high expectations for all)	1	2	3	4
	(circle one response)			
E Enjoyable — ENJOYABLE: Session was enjoyable and included a variety of activities:<br>• Play motivational music during exercise sessions<br>• Provide students with a variety of HIIT workout options<br>• Encourage students to reflect on their post exercise affect (i.e., how they are feeling)	1	2	3	4
Comments:				TOTAL /20

Note. Example strategies are listed below each of the SAAFE principles.

Supplemental Figure 2. B2L session observation checklist.
